# Identifying influenza-like illness presentation from unstructured general practice clinical narrative using a text classifier rule-based expert system versus a clinical expert

**DOI:** 10.1186/s12911-015-0201-3

**Published:** 2015-10-06

**Authors:** Jayden MacRae, Tom Love, Michael G. Baker, Anthony Dowell, Matthew Carnachan, Maria Stubbe, Lynn McBain

**Affiliations:** Patients First, Wellington, New Zealand; Sapere Research Group, Wellington, New Zealand; Department of Public Health, University of Otago Wellington, Wellington, New Zealand; Department of Primary Health Care & General Practice, University of Otago Wellington, Wellington, New Zealand; Compass Health, Wellington, New Zealand

## Abstract

**Background:**

We designed and validated a rule-based expert system to identify influenza like illness (ILI) from routinely recorded general practice clinical narrative to aid a larger retrospective research study into the impact of the 2009 influenza pandemic in New Zealand.

**Methods:**

Rules were assessed using pattern matching heuristics on routine clinical narrative. The system was trained using data from 623 clinical encounters and validated using a clinical expert as a gold standard against a mutually exclusive set of 901 records.

**Results:**

We calculated a 98.2 % specificity and 90.2 % sensitivity across an ILI incidence of 12.4 % measured against clinical expert classification. Peak problem list identification of ILI by clinical coding in any month was 9.2 % of all detected ILI presentations. Our system addressed an unusual problem domain for clinical narrative classification; using notational, unstructured, clinician entered information in a community care setting. It performed well compared with other approaches and domains. It has potential applications in real-time surveillance of disease, and in assisted problem list coding for clinicians.

**Conclusions:**

Our system identified ILI presentation with sufficient accuracy for use at a population level in the wider research study. The peak coding of 9.2 % illustrated the need for automated coding of unstructured narrative in our study.

## Background

Influenza and influenza like illness (ILI) are very important clinical conditions responsible for a high burden of global mortality and morbidity. Identification of the emergence and surveillance of these flu like syndromes, was and is, important [1–4] in order to implement appropriate health service response at both general practice and secondary hospital levels. Pandemic H1N1 influenza was detected in New Zealand in April 2009, and within two months spread widely across the country [5]. In New Zealand as in many other OECD countries General Practice and Primary care are responsible for the delivery of over 90 % of first line medical care, but are operated from small semi-autonomous units without a high degree of central control over clinical information recording and standardised coding systems. Community based services used a variety of response models. A research study was devised to acquire data on the pandemic and investigate the capacity for general practice to respond to the high demand for health care during the period.

Identification of patients with a particular condition is usually done using problem lists (disease classification data) or prescribed medicine lists [6, 7]. These lists are generated by clinicians during or shortly after a clinical encounter using the Read code classification system. Since the use of coded problem lists is highly variable between clinicians and between conditions [8], and we had no empirical evidence to suggest how comprehensively general practice had been classifying ILI, we could not rely solely on using this data set. We also suspected that prescribing of antiviral drugs (e.g., oseltamivir) would be an unreliable proxy, as it was not indicated in those solely presenting with ILI and some prescribing of it may have been prophylactic. Using software to classify clinical narrative has been postulated as a solution to such problems [9–12].

Our objective was to use automated software to identify ILI by classifying the unstructured clinical narrative written by physicians in community based care facilities. This approach has previously been successful in structured free text reports in an emergency department or hospital setting [10, 11, 13–21]. Little work has been done on information retrieval from community based care facilities and only a few papers have addressed searching narrative containing bespoke abbreviations or highly notational information [22]. Clinical narrative is traditionally difficult to process because it may often contain ambiguities, word compounds and a substantial body of synonyms to describe otherwise basic concepts. Because the clinical narrative in general practice is entered by a clinician using a keyboard during or shortly after a patient encounter it often contains typographical errors making the automation more difficult.

The aim of this paper is to describe the development and testing of a rule based expert-system to identify the presentation of ILI within general practice from routinely recorded clinical narrative. In doing so we outline a general approach towards algorithm development using text classifier processing that could be used in other clinical conditions where clinical coding is not universal.

## Methods

### System architecture

**Fig. 1:**
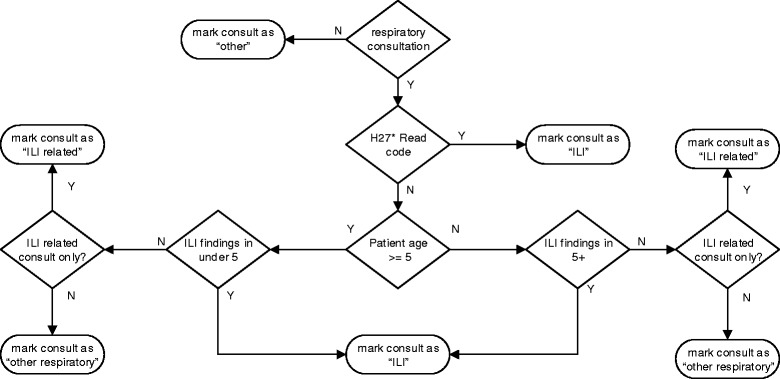
Clinical ILI algorithm schematic

We developed a clinical algorithm in conjunction with clinical experts based on each encounter’s narrative, problem lists and patient’s age. Its purpose was to classify the encounter into one of two states; the patient had influenza like illness or not. To aid training and understanding of the problem in more detail the negative state was sub-classified into three groups; influenza prophylactic; respiratory; and other. The detail of this can be seen in Fig. 1. Influenza prophylactic presentations were those where procedures were undertaken (e.g., flu vaccine) or medicines prescribed (e.g., antiviral prescription for travelling abroad) relating to influenza, but the patient did not present with an ILI. The negative state sub-classifications were not used in the final study analysis.

**Fig. 2:**
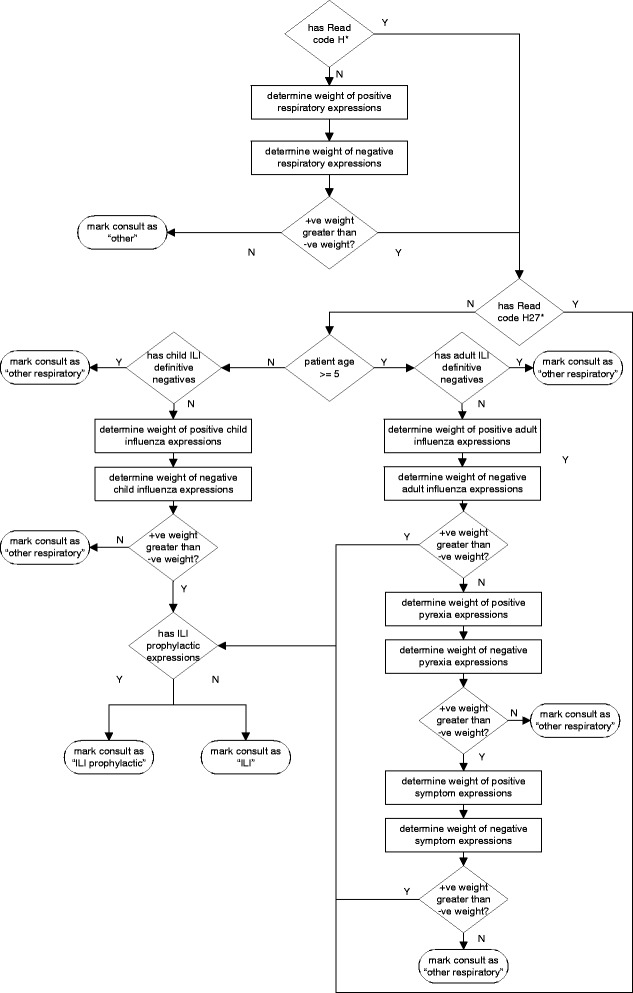
Software ILI algorithm schematic

The software inference algorithm (Fig. 2) is a more detailed implementation of the clinical algorithm (Fig. 1) which divides clinical decisions into distinct data level operations. It was developed in Microsoft.NET C#. It used a domain lexicon developed for identifying pertinent expressions that correspond to particular clinical observations within the algorithm. Clinical experts were responsible for compiling an initial list of pertinent signs and symptoms and other key terms that formed the lexical sets. Analysts were responsible for identifying synonyms and other potential key terms through the course of training rounds.

Each lexical set was coded into a heuristic dictionary using regular expressions, to account for abbreviations, spelling errors, synonyms and negations. Regular expressions were used to modify full and correct forms of words to make portions of words optional to match abbreviations, or to substitute similar phonetic characters to improve detection of misspelled words. For example the term “fever” used a regular expression of “fe?ve?r(([iey]sh)|y|s?)” to detect words stemmed from “fever” including “fevers”, “feverish” and “fevery” but to also account for abbreviations to a word stem of “fver”, “fevr” or “fvr” as well as misspellings such as “feverysh”. In this manner the algorithm sensitivity was increased by including more liberal character substitution expressions, while the specificity was decreased and vice versa. Matches to negation terms were controlled through the use of regular expression patterns, to both increase and decrease the appropriate word distances for particular terms which affected the measures of accuracy of the algorithm. The lexicon also included the concept of measurement-value pairs (e.g., “temp. 38.2” is analogous with a symptomatic description of pyrexia) used in other tools such as TOPAZ [14]. Sentence detection was used to limit the effect of negations and temporal references to the scope of each text chunk (clinical narratives are not always formed into proper grammatical structures in this case a sentence is more accurately described as related narrative). In this manner we applied a pipeline of rudimentary operations which included word stemming, sentence detection and chunking. Each square box in the flow diagram in Fig. 2 represents an NLP processing step; each step used a separate lexical set and parameters to achieve its goal.

Table 1 shows how the lexical sets are used within the algorithm. The number of separate expressions is shown as the expression count. Some terms are codified into multiple regular expressions depending on the complexity of the way in which those terms may be expressed. The purpose of providing a count in this manner is to provide some proxy indicator to the relative complexity of detection of terms within each set. In general negation based expressions had more terms, were longer and contained more complexity. This occurred because of the increased need to account for the relationship between pertinent expressions and negations. Examples of terms used within the lexical sets are shown in Table 2.

**Table 1:** Lexical sets

Lexical set	Description	Expressions
Adult Flu Definitive Negative Expressions	Expressions which relate specifically to other respiratory conditions in adults that are not ILI or that specifically negate ILI.	9
Adult Negative Flu Expressions	Expressions which relate to signs or symptoms that when negated decrease likelihood of ILI.	9
Adult Positive Flu Expressions	Expressions which relate specifically to signs or symptoms where present increase the likelihood of ILI.	9
Child Flu Definitive Negatives	Expressions which related specifically to other respiratory conditions in children that are not ILI or expressions which are specific negation of ILI itself.	10
Child Negative Flu Expressions	Expressions which relate to signs or symptoms in children that when negated decrease the likelihood of ILI.	25
Child Positive Flu Expressions	Expressions which related to signs or symptoms in children that would increase the likelihood of ILI if present.	30
Definitive Negatives	Expressions that relate to procedures associated with influenza, particularly prophylaxis but do not indicate ILI is currently present.	10
Negative Pyrexia Expressions	Expression that when either negated, or when present suggest a temperature that is not elevated.	16
Negative Respiratory Expressions	Expressions that relate to signs or symptoms that when negated decrease likelihood of respiratory disease.	28
Negative Symptom Expressions	Expressions that when present suggest an elevated temperature.	10
Positive Pyrexia Expressions	Expressions that related to signs or symptoms that may be associated with respiratory disease but not necessarily ILI.	11
Positive Respiratory Expressions	Expressions that related to signs or symptoms that when present, increase the likelihood that ILI is present.	36
Positive Symptom Expressions	Expressions which related to signs or symptoms that when present increase the likelihood of ILI.	10
ILI Prophylactic Expressions	Expressions that relate to influenza and contribute to workload in general practice but where there is no active disease process present in an individual. These expressions include those that related to the seeking of prophylactic measures for influenza.	10

**Table 2:** Examples of terms used within lexical sets

Expression type	Example terms
Flu	influenza, cold, muscle aches, cough, fever, URTI
Child Flu	influenza, cold, muscle aches, cough, fever, URTI
Flu Definitive Negatives	tonsillitis, asthma, ear check
Child Flu Definitive Negatives	otitis media, tonsillitis, asthma, ear check, pneumonia
Pyrexia	pyrexia, fever, hot, temperature 38-41
Respiratory	influenza, flu, ILI, H1N1, URTI, cough, runny nose, cold, phlegm, chesty, sore throat
Symptoms	cough, sore throat, red throat, myalgia
ILI Prophylactic	tamiflu, osiltamivir, flu vaccine

The tool applied two classes of logic for heuristic patterns. The first used logical disjunction to test for the existence of any appropriate expression. The disjunction was applied in a manner where the narrative was assessed against all terms in the lexical set with the presence of one or more terms resulted in a logical “true” output. The second used weights based on the proportion of pertinent expressions compared with negations. Each term present within the narrative for each lexical set was counted and contributed to the total weight of that lexical set for comparison against other lexical sets. This allowed for decisions within the algorithm to be made on the balance of the evidence.

The algorithm first identified if the patient had a respiratory condition by detecting an appropriate assigned Read code and then parsed the clinical narrative searching for expressions associated with respiratory illness and symptoms. If a patient was not coded with the more specific Influenza Read code (H27) the algorithm branched dependent on the patient’s age. The lexicons used differed for children and adults even in identifying the same types of symptoms. Any phrases that explicitly negated the presence of influenza immediately caused the algorithm to code the presentation as being “other respiratory” with no further checks. We used asymmetric word distance to limit the scope of negative and temporal phrases to pertinent expressions. Negative and temporal phrase scopes were limited to sentence chunks where sentences were detected through punctuation or end-of-line characters. Negation phrases included “no”, “not”, “nil”, “NAD” and permutations of these keywords. Regular expressions were formed to ensure that negation phrases were not detected within substrings of larger words. Temporal references included simple matches to keywords including “yesterday”, “prior”, “last time”, as well as detection of phrases of word pairs indicating a time period (“days”,”months”) matched with a reference to the past (“ago”, “previously”). For those older than five, checks were made for expressions that indicated a diagnosis or suspected influenza, presence of pyrexia and general symptoms associated with viral infection. For those younger than five years only a check was made for expressions related directly to influenza and fewer assumptions were made on more general symptomatic presentation. Separate lexical sets were used for some detection of child symptoms. The final step of the algorithm was designed to eliminate those that present for advice or prophylactic medicine only.

**Fig. 3:**
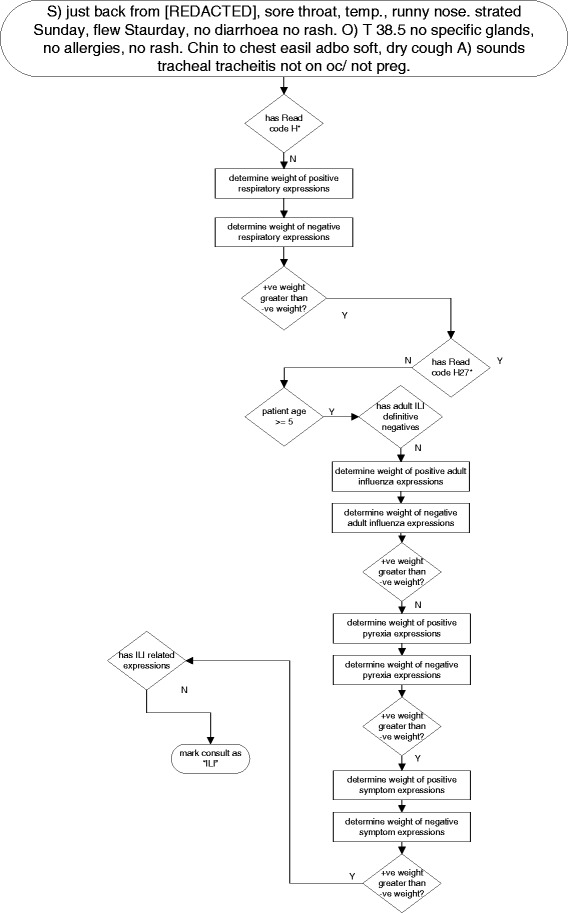
Software ILI algorithm applied to an example clinical narrative

How the algorithm parses a specific passage of text is illustrated in Fig. 3. It also provides an example of the typical format of a clinical narrative including descriptions of signs and symptoms and some of the abbreviations and typographical errors that can occur. It detects the presence of respiratory symptoms, does not find any statements explicitly ruling out ILI, finds observations indicating ILI, ascertains the patient has a high temperature, detects specific respiratory symptoms related to ILI and finally finds no evidence that the visit was only for discussion of ILI or for prophylactic treatment.

### Participants

All ninety-nine practices that were part of the primary health organisation (PHO) that collaborated on the research project were invited to participate in the study. Seventy-two practices agreed. The study data set included all patient encounters occurring from May to October, from 2007 to 2010 inclusive from participating practices. A total of 5.2 million routine encounter narrative records were included in the final study data set with associated problem lists, medications and disease screening activity information. Data was collected directly from patient management systems using standardised extraction scripts. No identifiable information was collected about any patient except the National Health Index unique identifier and all data used for the study was handled by the primary health organisation which routinely handles such data sets for contractual reporting purposes. The research team received no identifiable patient data. Proprietary software developed by the PHO was used to automate the extraction, packaging and secure transmission of the large data sets to the research team.

Demographics for patients funded in the participating practices over the study period were representative of all practices in the region. There appeared to be no substantial demographic factors that would be likely to contribute to any confounding within our results.

### Training

The algorithm was trained with a bias to promote specificity over sensitivity. Ten rounds of training were completed by a non-clinical analyst in conjunction with clinical experts. The training set consisted of six practices selected at random from all practices providing data for the research. One health care provider within each of these was selected at random to provide distinct sets of narrative. In total, 623 encounter narratives were used as the training set.

The training set was initially assessed by a clinical expert, following the agreed clinical algorithm. The assessor had access to the same data that the software algorithm could use, which included problem list data associated with the encounter, the content of the narrative and each patient’s age. Using the clinical algorithm they determined which category each presentation belonged within.

The goal of each training round was to improve the algorithm specificity, while maintaining or improving sensitivity. Because we predicted a relatively low incidence of ILI presentations based on local clinical experience consistent with international evidence [23, 24] we wished to keep the total number of false positive results to a minimum. The analyst compared the results of the software algorithm with the clinical assessor for each round. Results from each training round were analysed to identify portions of the heuristics and domain lexicon that were performing poorly. Training consisted of modifying the domain lexicon in conjunction with the clinical expert and modifying the pattern dictionaries to account for errors involving contextual discrimination, including temporal discrimination, finding validation and contextual inference as described by Chapman et al. [14].

### Testing

Eight practices were identified at random from the 72 providing data for the research. One provider in each of the eight practices was identified at random, and their clinical notes over a one week period were used as the test set. The six practices and providers whose notes were used as the training set were explicitly excluded from selection to eliminate any training bias. In total, 901 encounter narratives made up the test set.

The content of the test set was never used to inform the development or training of the clinical or software algorithm. It was used only once to determine final performance metrics and measures of accuracy.

### Gold standard

We considered the Gold standard to be the judgment of a clinical expert determining the presence of ILI on reading each clinical narrative. This was used to assess the performance of the algorithm during both training and test stages. A single clinical expert read each note, assessed the associated Read code information and classified each consult into an appropriate category using the algorithm as shown in Fig. 1. The expert was blinded from all results of the software algorithm.

### Ethics approval

This study was approved by the New Zealand Health & Disability Multi-region Ethics Committee under application number MEC/10/14/EXP.

## Results

**Fig. 4:**
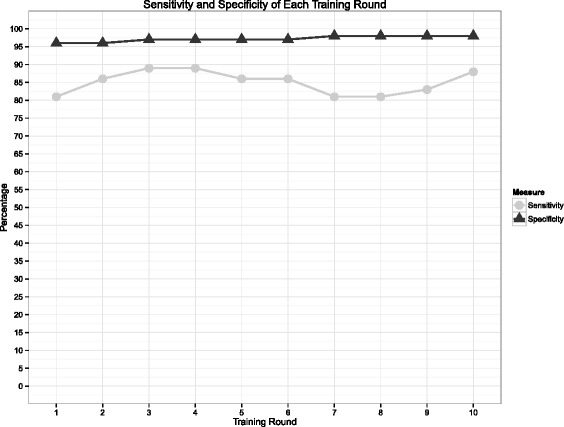
Algorithm sensitivity and specificity by training round

The results of the ten rounds of training of the algorithm can be seen in Fig. 4. While sensitivity was marginally higher during rounds 3 and 4 of training, the corresponding specificity at this point was not as high. The results of the tenth training round and final results of the test set can be seen in Tables 3 and 4. The algorithm performed similarly against the test set as the training set.

**Table 3:** Software algorithm performance versus gold standard in test and tenth-round training set

		Gold standard			
		Test		Training (round 10)	
	Has ILI	True	False	True	False
Algorithm	True	101	14	75	9
	False	11	775	10	529

**Table 4:** Software algorithm measures of performance in test and tenth-round training set

Measure	Training (round 10)	Test		
		Estimated value	95 % Confidence intervals	
			Lower limit	Upper limit
Incidence	0.136	0.124	0.103	0.148
Sensitivity	0.882	0.902	0.831	0.950
Specificity	0.983	0.982	0.970	0.990
Positive Predictive Value	0.893	0.878	0.804	0.930
F-measure	0.888	0.890	0.817	0.940

Binomial confidence limits were calculated using the method described by Collett et al. [25].

The performance of the final algorithm was pleasing with a mean classification rate of 7.3 clinical narratives per second. The algorithm was run on a physical server with a Dual Intel® Xeon® X5650 CPU running at 2.67 GHz with 24 Gb of RAM running Microsoft SQL Server 2008 R2.

When applied across all practices, the system detected substantially more presentations of ILI than were coded by clinicians. The monthly clinical coding rate of ILI by provider ranged from 0.3 to 9.2 % of the ILI encounters detected by the system. This represents a range of 8.9 %, showing there is little consistency in the rate clinicians code such encounters.

## Discussion

The rate at which ILI was coded in the problem list by GPs in their routine consultations was low. The peak coding rate for ILI in any month by any individual clinician was 9.2 % of that detected by the algorithm and meant that had we relied on clinical coding, our principal study would have significantly under reported the presentation of ILI. The range of individual clinician coding rate compared to detected ILI was 8.9 % and was so great that it would have been difficult to build a model based on observed problem list coding alone. Manually reviewing all five million records would have been impractical. The approach to use automated software to identify appropriate presentations therefore seems appropriate.

### Strengths and weaknesses

The results we have achieved are favourable in comparison to other studies. The specificity of the software matches the best seen in literature for classifying medical text [13, 26]. A recent rule-based approach to classifying influenza as a cause of death from death certificates reported higher PPV, substantially lower specificity and a slightly higher F-measure [12]. As a comparison, our task was likely more difficult due to the nature of clinician entered clinical narrative containing abbreviations, acronyms and typographical errors without well-formed grammar or structure. We had to consider the presentation of signs and symptoms beyond the mention of diagnoses. The rules we applied extended to those that addressed clinical reasoning and placing signs and symptoms into the context of a diagnosis without the necessary mention of the diagnosis itself. For these reasons we believe achieving similar measures of accuracy is a favourable result for this specific problem domain.

Training of the algorithm was focused on maximising specificity to minimise type II errors in our calculations to be conservative in our analysis of disease presentation. There were two keys to keeping our specificity high; the detection of expressions that unambiguously discounted ILI and those that negated otherwise pertinent findings. There is an existing body of literature on the latter from which we could draw [16, 27, 28]. Our system’s sensitivity is not as high as seen in other literature [13, 22], which suggests that more attention may need to be paid to identifying additional pertinent expressions or detecting the context in which they are used to include rather than negate them. Of course there is an inevitable trade-off between sensitivity and specificity and the balance within our algorithm may already be optimal. Our test method appears to be rigorous in the number of records we used, with narrow confidence intervals for the key parameters. A few prior studies use similarly large test datasets [13, 28], with one using a significantly larger test set [21] although this was not entirely classified by clinicians.

The gold standard here of clinician diagnosed ILI has limitations. The ILI case definition has evolved over time, with the current WHO surveillance case definition being an acute respiratory illness with onset during the last 10 days with history of fever or measured fever of ≥ 38.0C and cough [29]. Because this study was retrospective, it was not able to collect a complete set of data on each encounter sufficient to establish that the case did meet a standard ILI definition.

We used only one clinician to classify the test data. A previous study used overlapping sets across three clinicians, finding only 1 un-agreed result from 200 records [27]. Another using four clinicians disagreed on 12 from 200 records [28]. It is difficult to find clinicians with sufficient clinical experience, an appropriate analytical skill set and willingness to undertake such a tedious task. Given this and that little inter-rater disagreement has been previously found we believe that using a single clinician is appropriate as a ‘gold standard’ in this instance. We were conservative by using a high number of records for testing, and by minimising the introduction of bias by ensuring that the training sets were distinct from the test sets, and that test data were derived from a number of different clinicians.

Our results are satisfying considering the notational, varied and unstructured nature of the narrative we were attempting to classify. Based on our experience in this project of dealing with these narratives, the reported rate of 2 % spelling errors in medical notes [30] seems to be an underestimation for general practice in New Zealand. In general practice, clinicians are responsible for entering notes themselves, and this relies not only on their spelling ability and keyboard skills but also on their propensity to identify and correct such errors. Using heuristic pattern matching has allowed us to compensate for this potential source of error. Traditional Natural Language Processing does not cope well with notational narrative and requires abbreviation and notational expansion as a pre-analysis operation [22]. Although a large body of literature uses this approach it would likely have required a much larger computational demand and may have not been as successful. Natural Language Processing tends to be used to classify a wider range of presentations or diagnoses and operates on more formal medical texts, such as x-ray reports or discharge summaries that are often dictated and transcribed by medical typists, which would increase sentence structures and decrease abbreviations and spelling errors.

The problem domain we have addressed is unusual in focusing on community care facility data that is highly notational. One study has approached the classification of clinical narrative using clinician entered, notational data in an ophthalmology clinic [22], while another [21] used clinician entered data in an outpatient clinic but did not comment on the data structure or notational nature. Little literature uses routine general practice data for this purpose. The nature of general practice data is that there is a large variety in the presenting case-mix, with multiple problems arising in the course of one consultation making it difficult to use concepts such as primary reason for presenting. Although there are a multitude of systems used for academic purposes, very few are used in an operational medical environment and integrated with a clinical information system [31]. Our system is integrated with existing practice management systems commonly used in New Zealand general practice.

The use of regular expressions to provide a lightweight method of accounting for abbreviations and typographical errors may explain some of our success. Using regular expressions to account for common errors in words, particularly in the use of vowels and repeated consonants is easy and highly efficient. Breaking the NLP problem down into discrete steps, with separate lexicons and parameters appears to have simplified the task and allowed us to alter discrete parts of the algorithm without influencing others.

### Error analysis

Although our approach performed well in general there were some situations that proved difficult for our algorithm to address. The use of temporal modifiers referring to signs, symptoms and diagnoses were not detected. In most cases a reference to a past sign or symptom was not relevant to the current presentation. This is particularly relevant for acute disease such as those that present as ILI. In longer term disease past signs and symptoms may be more useful in determining a final diagnosis. Clinical narratives at times contained references to signs, symptoms and diseases belonging to the subject’s relatives, such a parent or sibling. This was more often associated with clinical narratives for children. The algorithm had difficulty distinguishing between signs or symptoms reported about the subject by a relative, or whether the signs and symptoms were those reported by the subject about a relative with the later contributing to type I errors.

The algorithm detected the negation of pertinent terms but was confused by particularly wordy or convoluted negation phrases e.g., “No obvious or real cough” or “No cough in the past few weeks but has started in the last day”. It is likely that an improved part-of-speech tagging approach may address these limitations.

### Future work

Current influenza surveillance is carried out in general practice in New Zealand by paper based manual systems or on manual problem list coding by clinicians [5]. Paper based systems can only be deployed to a limited number of practices, and suffer the same issue of relying on clinicians to remember to report cases of interest. Paper based systems suffer from a latency caused by the delays in completing paper work, transmission and collation. Existing electronic systems report on items recorded within problem lists. Our results show that at best, this type of reporting detects a small proportion of real cases, and the range of the rate makes it difficult to model and extrapolate such figures from manual recording alone.

The early detection of disease outbreaks can be an important factor in the ability of public health services to successfully intervene [32, 33]. Text classifying expert systems have the potential to be used for large scale surveillance of presentation of ILI to general practice, or to other care settings where medical narrative is available electronically with a high resolution on a daily basis. Situating such an automated alerting or reporting system within general practice offers minimal latency of outbreak detection. The research team is currently developing this concept further.

This method could be scaled to be used across an even larger set of patient records in near-real time. Our approach is not computationally onerous and could be implemented within existing practice management systems, running on modest desktop computing equipment to aid in an increased rate of problem list coding. We ensured that the training and test records sets contained no cross-over between clinicians or facilities contributing to each which provides some demonstration that the algorithm is generally applicable beyond the records which were used to develop and train it. No single clinician or facility contributed more than 7 % of overall records for training or test purposes. Because of the small number of medical training facilities in New Zealand (two medical schools and a single vocational training organisation) we expect the variation between clinicians in the use of pertinent expressions and key semantics of note taking to be represented in the study group. Currently clinicians must manually find and select appropriate problem list codes to classify encounters within the mainstream patient management systems in general practice in New Zealand. This process contributes to the highly variable rate of coding across clinicians and conditions. Providing feedback, incentives and evidence based guidelines have been shown to improve data quality in clinical coding [34] and national and regional initiatives have done just this for long term conditions, but with little emphasis on the classification of acute presenting problems. A rule based approach could process narrative notes in real time, and suggest items of interest for coding with high accuracy. This approach has the potential to improve the coding of less significant or short-term problems [35]. Maintaining accurate and comprehensive problem lists can be useful to general practices who wish to keep a complete health record for their patients and manage them using this data. The limitation of this approach is that the algorithm development is based on specific target conditions each of which would have their own particular clinical presentations.

We could improve our approach by having the software output and store meta-data about the performance of each narrative and how it is branched through the algorithm to reach a final classification. This would likely help with training the algorithm and understanding how particular sub-components of the algorithm are performing which in turn would lead to better final performance.

## Conclusions

This paper describes the successful use of a heuristic rule-based expert system for identifying presentations of ILI in general practice from routine clinical narrative. The system performed to a standard sufficient to use for population based research similar to the best performing systems documented for other similar problems.

This research demonstrates that a text classifier can be applied successfully to a clinical narrative that is highly abbreviated and contains substantial spelling and typographical errors. General practice captures a large volume of medical information in narrative form on patients and the application of the techniques described can unlock this previously difficult to access information source. Furthermore this type of approach is computationally simple enough to apply to millions of records or in real time in general practice surgeries.

We have discovered that the clinical coding rate for ILI over the period of the study was very low, necessitating the use of such a tool for our specific research study. These results highlight an important finding for researchers that the routine coding of acute conditions such as ILI within general practice may be substantially lower than the actual presentation. The system also has potential to be used for automated on-going disease surveillance.
